# A potential allosteric inhibitor of SARS-CoV-2 main protease (M^pro^) identified through metastable state analysis

**DOI:** 10.3389/fmolb.2024.1451280

**Published:** 2024-09-06

**Authors:** Asma Fatima, Anupriya M. Geethakumari, Wesam S. Ahmed, Kabir H. Biswas

**Affiliations:** Division of Biological and Biomedical Sciences, College of Health and Life Sciences, Hamad Bin Khalifa University, Qatar Foundation, Doha, Qatar

**Keywords:** COVID-19, allosteric regulator, MD simulation, metastable states, M^pro^, ZINC15

## Abstract

Anti-COVID19 drugs, such as nirmatrelvir, have been developed targeting the SARS-CoV-2 main protease, M^pro^, based on the critical requirement of its proteolytic processing of the viral polyproteins into functional proteins essential for viral replication. However, the emergence of SARS-CoV-2 variants with M^pro^ mutations has raised the possibility of developing resistance against these drugs, likely due to therapeutic targeting of the M^pro^ catalytic site. An alternative to these drugs is the development of drugs that target an allosteric site distant from the catalytic site in the protein that may reduce the chance of the emergence of resistant mutants. Here, we combine computational analysis with *in vitro* assay and report the discovery of a potential allosteric site and an allosteric inhibitor of SARS-CoV-2 M^pro^. Specifically, we identified an M^pro^ metastable state with a deformed catalytic site harboring potential allosteric sites, raising the possibility that stabilization of this metastable state through ligand binding can lead to the inhibition of M^pro^ activity. We then performed a computational screening of a library (∼4.2 million) of drug-like compounds from the ZINC database and identified several candidate molecules with high predicted binding affinity. MD simulations showed stable binding of the three top-ranking compounds to the putative allosteric sites in the protein. Finally, we tested the three compounds *in vitro* using a BRET-based M^pro^ biosensor and found that one of the compounds (ZINC4497834) inhibited the M^pro^ activity. We envisage that the identification of a potential allosteric inhibitor of M^pro^ will aid in developing improved anti-COVID-19 therapy.

## Introduction

The COVID-19 pandemic has tremendously affected human health and economic activities around the world (Cucinotta and Vanelli, 2020; Lamers and Haagmans, 2022; Wu et al., 2020). As of 17 March 2024, it has caused more than seven million deaths and 774 million infections, overwhelming the healthcare system even in countries with the best healthcare setup (WHO Coronavirus (COVID-19) Dashboard | WHO Coronavirus (COVID-19) Dashboard With Vaccination Data) (Miller et al., 2020; Cooper et al., 2023; Salje et al., 2020). The restrictions implemented to control the pandemic have resulted in substantial economic losses globally, possibly increasing losses due to the disease’s long-term impact on patient health (Jackson and Overview, 2022). At the heart of the problem is the high infectivity and rapid transmission of SARS-CoV-2 (Bian et al., 2021; Tao et al., 2021a). The viral infection cycle begins with the binding of the virus to receptors expressed on human host cells, leading to the internalization of the virus and release of its RNA genome into the host cell cytosol (V’kovski et al., 2021). This is followed by the translation of viral RNA into polyproteins containing non-structural proteins (NSPs) critical for the viral replication complex formation (Ludwig and Zarbock, 2020; Cevik et al., 2020; Harrison et al., 2020; Khalaf et al., 2020). However, to perform their function, these NSPs need to be cleaved into individual proteins through the proteolytic activity of the two SARS-CoV-2 proteases, the main protease (M^pro^; chymotrypsin-like protease; 3CL^pro^; NSP5) (Jin et al., 2020; Hu et al., 2022; Legare et al., 2022) and papain-like protease (PL^pro^; NSP3) (Harrison et al., 2020)^,^ (Lee et al., 2022; Trougakos et al., 2021; Vicenti et al., 2021).

The rapid development of vaccines has indeed decelerated COVID-19-related death rates (Huespe et al., 2023; Golob et al., 2021; Hall et al., 2022; Robson, 2020). However, the continued emergence of SARS-CoV-2 variants with increased infection potential, disease severity, and resistance to antibody-mediated neutralization could make the currently administered vaccines ineffective (Jangra et al., 2021; Uwamino et al., 2022; Chen et al., 2022; McLean et al., 2022; Mistry et al., 2022; Bian et al., 2021; Tao et al., 2021a). Additionally, the emergence of novel infectious strains of coronaviruses remains a constant possibility (Tao et al., 2021a). Therefore, pharmacological targeting of SARS-CoV-2 proteases, among other essential viral proteins, remains a viable alternative for combating the pandemic (Schütz et al., 2020; Chiou et al., 2021; Jan et al., 2023a; Cheohen et al., 2023).

Given the importance of M^pro^ in SARS-CoV-2 replication and infection, several inhibitors have been developed so far (Jin et al., 2020; Günther et al., 2021; Samrat et al., 2022; Pang et al., 2023; Chan et al., 2021; Sabbah et al., 2021; Narayanan et al., 2022; Shree et al., 2022; Ge et al., 2022; Huff et al., 2022; Rossetti et al., 2022). These include competitive inhibitors such as TDZD-8 (Jin et al., 2020), ebselen (Jin et al., 2020), N3 (Arafet et al., 2020), 11a (Dai et al., 2020), and α-ketoamide (13b) (Zhang et al., 2020) that inhibit the activity of the protein by binding to its catalytic site. More importantly, the M^pro^ inhibitor nirmatrelvir, which is a part of Paxlovid^®^, has been approved by the FDA as a SARS-CoV-2 antiviral drug (Paciaroni et al., 2023). However, the competitive inhibition of M^pro^ places the catalytic site under constant evolutionary pressure to evolve in order to accommodate the substrate peptide into the catalytic site and proteolytic cleavage (Wenthur et al., 2014). This is especially relevant in the case of SARS-CoV-2 as its RNA genome is prone to mutations and the virus has already been reported to have evolved into many variants (Callaway, 2022; Tao et al., 2021b). Therefore, any mutation in the M^pro^ catalytic site may reduce the efficacy of antiviral drugs such as nirmatrelvir. Mutations of catalytic site residues, such as Q189K and G143S, have been reported to reduce nirmatrelvir efficacy (Noske et al., 2023). Therefore, it is necessary to continue SARS-CoV-2 antiviral drug discovery research with alternate approaches such as the development of allosteric modulators to regulate M^pro^ activity (Tee and Berezovsky, 2024).

Allosteric modulation or allostery can be defined as the regulation of the protein activity by the interaction of a ligand to a site that is distinct from the catalytic or substrate-binding site of the protein (Biswas, 2017; Astore et al., 2024; Wu et al., 2024; McCullagh et al., 2024). The ligand binding-induced allosteric modulation can be either activating or inhibitory depending on the nature of the impact of the allosteric ligand binding on the structure and structural dynamics of the protein (Biswas, 2017; Biswas K. et al., 2008; Biswas and Visweswariah, 2011; Biswas et al., 2015). For instance, an inhibitory allosteric ligand can induce conformational changes in the catalytic site or change the structural dynamics of the catalytic site in a way that stabilizes the protein in its inactive form (Bhat et al., 2022b; Yuce et al., 2021). Some previous studies have reported allosteric inhibitors that bind to allosteric sites in M^pro^ (Günther et al., 2021; Strömich et al., 2022; Tao X. et al., 2021; Verma and Pandey, 2021; Jiménez-Avalos et al., 2021). However, most of these allosteric inhibitors were identified through *in silico* docking studies using FDA-approved drugs, natural products, and small drug libraries (Bhat et al., 2022b; Yuce et al., 2021; Tumskiy et al., 2023).

The M^pro^ monomer is structurally divided into three domains: domain-I (10–96) and domain-II (102–180) constitute the catalytic pocket, while domain-III (200–303) is a C-terminal α-helical domain linked to domain-II by a linker loop (183–197) (Jin et al., 2020). The catalytic dyad residues (H41 and C145) lie between domain-I and domain-II (Jin et al., 2020). Additionally, electrostatic and hydrophobic interactions between domain-III of two monomers enable M^pro^ homodimerization, which is critical for its proteolytic activity (Paciaroni et al., 2023; Ferreira et al., 2022). Mutation-induced variations in M^pro^ structural dynamics have been shown to alter kinetic parameters such as *K*_m_ and the catalytic efficiency (*k*_cat_ */K*_m_) of the enzyme (Chen et al., 2023). For instance, the V186F (7.2% in the gamma variant) and A260V (3.7% in alpha and 5% in delta variants) M^pro^ mutants showed an increase in their *k*_cat_ */K*_m,_ while two of the highly prevalent mutants K90R (99.8% in beta variant) and P132H (99.9% in omicron) showed an increase in the *K*_m_ and a decrease in their catalytic efficiency (*k*_cat_ */K*_m_) (Chen et al., 2023). However, the K90RP132H double-mutant showed the opposite (a decrease in *K*_m_ and an increase in *k*_cat_ */K*_m_) results compared to the single mutants (Chen et al., 2023). Furthermore, several M^pro^ mutations have been reported to affect M^pro^ thermal stability (Chen et al., 2023). For instance, structural analysis of the P132H mutant M^pro^ revealed that the residue H132 allosterically enhances the dynamic flexibility of the catalytic pocket’s entry site, which resulted in a reduction in the thermal stability of this mutant (Bhat et al., 2024). Carli et al. (2021) reported that the accessibility of the M^pro^ catalytic dyad residues is also correlated with some key interactions that are distant from the catalytic site, such as the interaction of residue E47 with residue L57 and the interaction of residue Y118 with residue N142 (Carli et al., 2021). Additionally, these allosteric interactions were present in some of the M^pro^ metastable states and absent in others (Carli et al., 2021). This may also suggest differences in the proteolytic activity among various M^pro^ metastable states (Carli et al., 2021). Furthermore, structural analysis of the M^pro^ metastable states revealed the formation and deformation of certain pockets relative to the catalytic site structure in some of the metastable states (Carli et al., 2021). This raises the possibility of targeting these potential allosteric interactions in M^pro^ using chemical ligands to inhibit its proteolytic activity and, thus, develop an anti-SARS-CoV-2 drug.

In the present study, we intended to identify metastable states of M^pro^ that exhibit (i) a deformed catalytic site and (ii) a well-formed allosteric site that could be targeted using chemical ligands. For the identification of this metastable state, we analyzed the binding affinity of metastable states to the M^pro^ N-terminal auto-cleavage peptide (TSAVLQSGFRK) (Xue et al., 2008) and selected two (amongst a total of 18) M^pro^ metastable states, namely, m2_c5 and m1_c11, that showed decreased binding affinity to the substrate peptide. A comparative analysis of the catalytic sites of the M^pro^ metastable states m1_c11 and m2_c5 with a reference M^pro^ X-ray crystal structure (PDB: 6Y84; https://www.rcsb.org/structure/6Y84) revealed that the M^pro^ metastable state m2_c5 has a deformed catalytic pocket. Subsequently, we analyzed its structure to identify potential allosteric sites that show high druggability. Following the identification of the potential allosteric sites, we performed an *in silico* screening of a library of 4.2 million drug-like compounds obtained from the ZINC15 database (Irwin and Shoichet, 2005), targeting the potential allosteric sites, and validated high-affinity binders through additional, blind computational docking and molecular dynamics (MD) simulation analysis. Finally, *in vitro* assays using our in-house BRET-based M^pro^ biosensor (Geethakumari et al., 2022a) revealed that one of the three high-affinity allosteric site binders inhibited M^pro^ activity with micromolar affinity.

## Materials and methods

### Protein–peptide docking

The M^pro^ N-terminal auto-cleavage peptide, TSAVLQSGFRK, was docked on the catalytic site of all 18 M^pro^ metastable states (Carli et al., 2021) using AutoDockFR (Ravindranath et al., 2015) (ADFR) software suite, a docking software application for flexible receptor and ligand docking. For this, we took advantage of the M^pro^ metastable states reported by Carli et al. (2021) recently. In brief, the authors analyzed a total of 20,000 conformers of M^pro^ obtained from a 100-µs-long MD simulation (D. E. Shaw Research, “Molecular Dynamics Simulations Related to SARS-CoV-2,” D. E. Shaw Research Technical Data, 2020. https://www.deshawresearch.com/downloads/download_trajectory_sarscov2.cgi/) (Carli et al., 2021) of a dimeric M^pro^ structure (10,000 conformers from each monomer) and identified 18 metastable states (11 metastable states from one monomer and 7 metastable states from the second monomer of the M^pro^ dimer). The peptide coordinates used for docking with ADFR were obtained from the peptide-M^pro^ complex structure (PDB: 2Q6G) (Xue et al., 2008). The output of protein–peptide docking consisted of the binding energies and binding pose of the docked peptide on the M^pro^ metastable states. The docked complexes were ranked based on their binding energies, and the high-affinity binding poses of the M^pro^ N-terminal auto-cleavage peptide with M^pro^ metastable states were analyzed using PyMOL (WL, 2002; Yuan et al., 2017).

### M^pro^ allosteric site prediction

From protein–peptide docking, we selected the M^pro^ metastable states m1_c11 and m2_c5, one from each monomer of the M^pro^ dimer, with the lowest binding affinity for the M^pro^ N-terminal auto-cleavage peptide. Next, we performed a structural analysis of the catalytic sites of the two M^pro^ metastable states (m1_c11 and m2_c5) against the reference structure (PDB: 6Y84; https://www.rcsb.org/structure/6Y84) using PyMOL (WL, 2002; Yuan et al., 2017). The primary goal of this structural analysis was to identify the M^pro^ metastable state with the most significant structural deformation, which leads to the identification of the M^pro^ metastable states m2_c5 that showed major structural variations compared to the reference structure. Thereafter, we utilized the structure of the M^pro^ metastable state m2_c5 for the identification of potential allosteric sites using the DoGSiteScorer (Volkamer et al., 2012) software application, which is an automated pocket detection tool available on the ProteinsPlus server (https://proteins.plus/help/dogsite). The identified potential allosteric sites were then further analyzed using PyMOL (WL, 2002; Yuan et al., 2017) and were used for *in silico* screening of a library of drug-like molecules for the identification of potential M^pro^ allosteric site binders.

### Ligand library preparation

The ZINC15 chemical compounds library of approximately 4.2 million was obtained from the ZINC15 database website with the tranches specification of 3D representation, standard reactivity, in stock, LogP value in the range of 2–4.5, and molecular weight ranging from 400 to 500 Da (https://zinc.docking.org/tranches/home/) (Irwin and Shoichet, 2005). The chemical compound library was downloaded in the PDBQT format and was used in the same format for *in silico* chemical compound library screening (Sterling and Irwin, 2015).

### *In silico* screening

In order to identify potential M^pro^ allosteric site binders, we screened a library of approximately 4.2 drug-like chemical compounds obtained from the ZINC15 chemical compound library (Irwin and Shoichet, 2005). To screen the compounds, we performed a site-specific docking of the chemical compounds on the identified allosteric site in the M^pro^ metastable state m2_c5 using idock software (Li et al., 2012), which is a faster version of AutoDock Vina (Trott and Olson, 2010) and, thus, requires reduced computational resources. Ligands were ranked according to their predicted binding energies, and the top 400 compounds showing high energy binding to the potential allosteric site in the site-specific docking screen were further screened through blind docking using AutoDock Vina (Trott and Olson, 2010) software for their preference to bind to the identified allosteric sites in the M^pro^ metastable state m2_c5 structure. These led to the identification of three compounds, namely, ZINC11696924, ZINC12383815, and ZINC4497834, that were predicted to bind to the M^pro^ metastable state m2_c5 with high affinity and with a preference for the identified potential allosteric sites.

### *In silico* physiochemical, ADME, and druglikeness prediction

To determine the efficacy and safety of the predicted potential M^pro^ allosteric binders ZINC11696924, ZINC12383815, and ZINC4497834, we determined their physicochemical properties, ADME, and druglikeness. These included features such as molecular weight, number of heavy atoms, aromatic heavy atoms, rotatable bonds, hydrogen-bond acceptors, hydrogen-bond donors, and topological polar surface area (TPSA), as well as pharmacokinetic properties like gastrointestinal absorption (GI absorption), blood–brain barrier permeability, P-glycoprotein substrate status, interactions with cytochrome P450 enzymes, and druglikeness as per Lipinski’s rule of five, the Ghose filter, Veber rules, and the bioavailability score. These parameters were predicted using the web tool SwissADME (http://www.swissadme.ch/) (Daina et al., 2017).

### MD simulation and analysis

MD simulations of the M^pro^ metastable state m2_c5 in the apo and in complex with high-affinity allosteric binders (ZINC11696924, ZINC12383815, and ZINC4497834) were performed using NAMD 2.13 software (Phillips et al., 2005) using the CHARMM36 force field (Huang et al., 2017), largely as described previously (Geethakumari et al., 2022a; Jan et al., 2023b; Ahmed et al., 2022; Philip et al., 2023; Altamash et al., 2021; Uddin et al., 2024; Ahmed et al., 2024; Arshad et al., 2023). The topology and parameter files for the simulation were generated using the CHARMM-GUI online server (Jo et al., 2008). First, the complex was dissolved in an explicit solvent, employing the TIP3P cubic water box (Jorgensen et al., 1983). The box had a minimum distance of 10 Å between its edges and any of the atoms in the complex. Subsequently, 0.15 M NaCl was added to the solvated system. The simulation system had 149,117, 149,196, 149,199, and 149,187 atoms for the M^pro^ metastable state m2_c5 in apo and in complex with ZINC12383815, ZINC11696924, and ZINC4497834, respectively. Before the production run, the biomolecular simulation system was taken through energy minimization, thermal annealing, and equilibration, applying periodic boundary conditions as previously described. Subsequently, three independent 100 ns production simulations were run, with a time step of 2 fs, and trajectory frames were saved every 10,000 steps. For handling short-range non-bonded interactions, a 12 Å cut-off with a 10-Å switching distance was utilized. As for long-range non-bonded electrostatic interactions, the particle-mesh scheme at a 1 Å PME grid spacing was employed (Feller et al., 1995; Steinbach and Brooks, 1994; Essmann et al., 1995). Trajectory analysis was performed using the available tools in visual molecular dynamics (VMD) (Humphrey et al., 1996), including Cα atom root-mean-square deviation (RMSD) and root-mean-square fluctuation (RMSF) measurements. The free energy change of binding was estimated using the molecular mechanics Poisson–Boltzmann surface area (MM-PBSA) method (Kollman et al., 2000). This was achieved using the CaFE 1.0 (Liu and Hou, 2016) plugin in VMD (Humphrey et al., 1996). Hydrogen bond analysis was performed with a cut-off distance of 3.5 Å and an A-D-H angle of 20° using the “Hydrogen Bonds” analysis plugin in VMD (Humphrey et al., 1996). Dynamic cross-correlation (DCC) analysis based on the position of Cα atoms was performed using MD-task (Brown et al., 2017), a python script suite.

### Expression and purification of SARS-CoV-2 M^pro^

The BL21-CodonPlus (DE3) *E. coli* strain was chemically transformed with the SARS-CoV-2 M^pro^ bacterial expression plasmid, pETM33_NSP5_M^pro^ (Addgene #156475) (Mihalic et al., 2023). Transformed bacteria were grown in Luria broth (LB) media containing 50 μg/mL kanamycin and chloramphenicol overnight at 180 rotations per minute (rpm) and 37°C. The inoculum was transferred to the fresh LB media and incubated for 2 h at 37°C and 220 rpm, followed by protein expression induction using IPTG at a final concentration of 1 mM for 2.30 h at 37°C and 220 rpm. The cells were pelleted at 4,000×g at 4°C for 10 min and re-suspended in 10 mL lysis buffer [50 mM Tris (pH 8), 300 mM NaCl, 10 mM β-mercaptoethanol (β-ME), 1 mM PMSF, and 10% (v/v) glycerol], followed by sonication on ice for 15–30 min and centrifugation at 4,000× g at 4°C for 10 min. The supernatant was collected and centrifuged at 18,000×g at 4°C for 1 h. The supernatant was incubated with the GSH beads for 2 h, followed by washing with a buffer containing 50 mM Tris (pH 7), 150 mM NaCl, 10 mM β-mercaptoethanol (β-ME), 1 mM EDTA, 10% (v/v) glycerol, and 0.01% TritonX-100. The GSH beads containing M^pro^ were incubated with PreScission Protease (GE Healthcare # 27–0843–01) in cleavage buffer [50 mM Tris (pH 7), 150 mM NaCl, 1 mM EDTA, 1 mM DTT, 10% (v/v) glycerol, and 0.01% TritonX-100] for 16 h at 4°C. The supernatant containing SARS-CoV-2 M^pro^ obtained after centrifugation at 4°C and 500×g was aliquoted and stored at −80°C until further usage.

### Cell culture and M^pro^ biosensor lysate preparation

To obtain cell lysates containing the M^pro^ biosensor for use in *in vitro* M^pro^ assays, HEK 293T cells were transfected with the pmNG-Mpro-Nter-auto-NLuc (Geethakumari et al., 2022b) plasmid DNA using polyethyleneimine (PEI) transfection, as described previously (Ahmed et al., 2024; Uddin et al., 2024; Altamash et al., 2021), (Biswas and Visweswariah, 2011), (Biswas et al., 2015), (Ahmed et al., 2024; Uddin et al., 2024; Altamash et al., 2021), (Biswas K. H. et al., 2008; Moovarkumudalvan et al., 2022; Geethakumari et al., 2022b). Cells were washed with cold Dulbecco’s phosphate-buffered saline (DPBS) 48 h post-transfection, and cells were lysed in a buffer containing 50 mM HEPES (pH 7.5), 50 mM NaCl, 0.1% Triton-X 100, 1 mM DTT, and 1 mM ethylenediamine tetraacetic acid (EDTA) (Grum-Tokars et al., 2008). Cell lysates were collected in a 1.5-mL Eppendorf tube and centrifuged at 4°C for 1 h at 14,000 rpm. The supernatant was collected and stored at −80°C for later usage.

### *In vitro*, BRET-based M^pro^ proteolytic cleavage assay

The allosteric inhibitors at various concentrations (ranging from 10^–4^ to 10^–13^ M) were incubated with 2 μM of recombinantly purified SARS-CoV-2 M^pro^ for 2 h at 37°C in a buffer containing tris-buffered saline (TBS), 0.6 M sodium citrate, 1 mM EDTA, and 2 mM DTT, followed by the addition of cell lysates prepared from HEK293T cells expressing the M^pro^ biosensor (Geethakumari et al., 2022b). GC376 (GC376 sodium; AOBIOUS-AOB36447; stock solution prepared in 50% DMSO at a concentration of 10 mM) served as the control. Bioluminescence resonance energy transfer (BRET) measurements were performed at 37°C by the addition of furimazine (Promega, Wisconsin, United States) at a dilution of 1:200. The bioluminescence measurements at 467 and 533 nm were recorded using a Tecan SPARK multimode microplate reader and used to calculate BRET ratio (533 nm/467 nm). BRET ratios, obtained after 30 min of the initiation of the cleavage reaction through the addition of recombinant M^pro^ either in the absence or in the presence of increasing concentrations of the compounds, were fit to a sigmoidal dose–response curve to determine the IC_50_ values.

### Data analysis and figure preparation

The output files from protein–peptide docking and protein–ligand docking were analyzed using a molecular visualization tool, PyMOL (Molecular Graphics System, Version 2.5.2, Schrödinger, LLC; pymol. org) (WL, 2002). For figure preparation, images were exported from PyMOL (WL, 2002) in a ray-traced, transparent background PNG format. Inkscape (Inkscape-1.1 version open-source software licensed under the GPL) and Microsoft PowerPoint software were used for assembling figures. GraphPad Prism (GraphPad Software, La Jolla, California, United States; www.graphpad.com), in combination with Microsoft Excel, was used for data analysis and graph preparation.

## Results and discussions

### Identification of an M^pro^ metastable state for potential allosteric targeting

**FIGURE 1:**
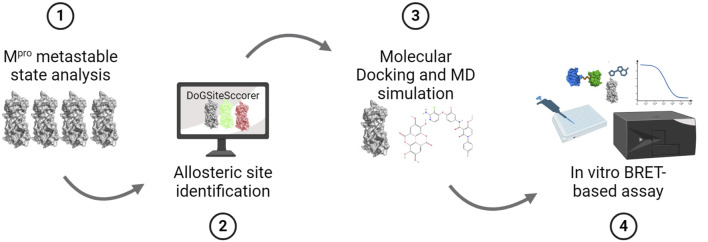
SARS-CoV-2 M^pro^ allosteric inhibitor discovery pipeline. Schematic representation showing the stepwise methodology employed in this study that includes (1) analysis of the M^pro^ metastable states to identify those that contain a deformed catalytic site such that it cannot bind the substrate peptide, (2) identification of allosteric sites in the selected M^pro^ metastable state, (3) performing *in silico* drug screening to identify potential ligands for the identified allosteric sites and perform MD simulation of top-scoring ligands to determine the stability of their binding, and (4) performing *in vitro* M^pro^ cleavage assay using a BRET-based biosensor to determine the inhibitory potency of identified allosteric site ligands.

In order to identify an allosteric site in the M^pro^ structure, we first attempted to identify a M^pro^ metastable with a deformed catalytic site such that it can no longer bind to its cleavage substrate peptides (Figure 1). For this, we took advantage of the structural analysis performed on a 100-µs-long MD simulation trajectory of M^pro^ reported by Carli et al. (2021). In brief, the authors analyzed a total of 20,000 configurations of M^pro^ obtained from the 100 µs-long MD simulation trajectory (D. E. Shaw Research, “Molecular Dynamics Simulations Related to SARS-CoV-2,” D. E. Shaw Research Technical Data, 2020. https://www.deshawresearch.com/downloads/download_trajectory_sarscov2.cgi/) (Carli et al., 2021) of a dimeric M^pro^ structure (10,000 configurations from each monomer) and identified a total of 18 metastable states (11 from monomer 1 and 7 from monomer 2; monomers were referred to as either m1 or m2). This high-dimensional analysis involved the calculation of the ψ backbone-dihedral distance and the mobile contact map distance to monitor variations in the protein backbone and the side chain rearrangements, respectively. The selected configurations, based on ψ backbone-dihedral distance and the mobile contact map distance, were used for free energy estimation. Furthermore, the authors defined a metastable state as a group of configurations with similar free energy minima that persisted for tens of nanoseconds throughout the 100-µs-long MD simulation trajectory (Carli et al., 2021).

**FIGURE 2:**
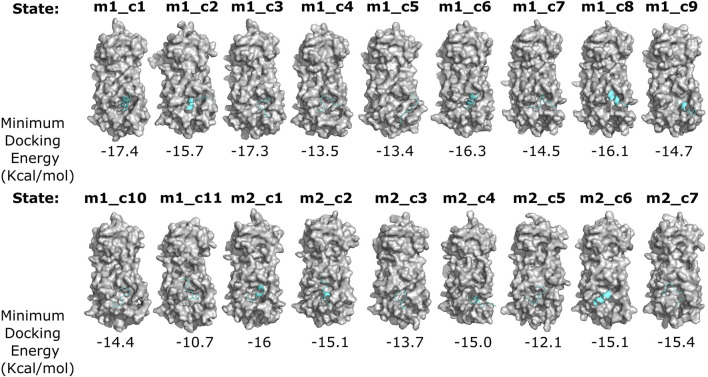
Differential binding affinity of the N-terminal M^pro^ autocleavage site peptide for various M^pro^ metastable states. Surface representation of the indicated M^pro^ metastable states (gray) and docked N-terminal autocleavage site peptide (cyan) obtained from flexible receptor–ligand docking analysis using the ADGFR suite (Ravindranath et al., 2015). Minimum docking energies (for the top-scoring poses) are indicated below each complex. Note the differential binding modes, secondary structure, and binding energies of the N-terminal autocleavage site peptide with various M^pro^ metastable states.

To identify an M^pro^ metastable state with a deformed catalytic site, we performed protein–peptide docking using ADFR software (Ravindranath et al., 2015) to examine the binding affinity of all 18 metastable states for the M^pro^ N-terminal autocleavage substrate peptide (TSAVLQSGFRK) (Xue et al., 2008). We posited that differences in the binding energies, determined through the docking study, would provide insights into the differences in the catalytic site structures in these metastable states (Ferreira et al., 2021). These docking simulations revealed two metastable states, namely, m2_c5 and m1_c11, that showed markedly reduced predicted binding affinities for the M^pro^ substrate peptide (−12.1 and −10.7 kcal/mol, respectively) compared to that of all other metastable states (values ranging from 13.4 to 17.4 kcal/mol) (Figure 2).

**FIGURE 3:**
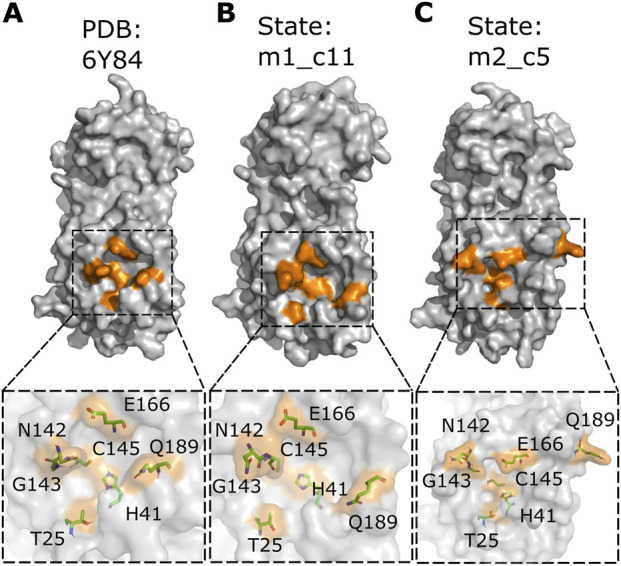
Altered position and orientation of catalytic site residues in the M^pro^ metastable states m1_c11 and m2_c5. **(A–C)** Surface representation of the M^pro^ crystal structure (PDB: 6Y84; https://www.rcsb.org/structure/6Y84) **(A)** and metastable states m1_c11 **(B)** and m2_c5 **(C)** highlighting the catalytic site residues in orange. Outsets show the location and orientation of the catalytic site residues (both side chain and main chain atoms) in the stick representation in the indicated complexes. Note the prominent alteration in the position of catalytic site residue Q189 in the M^pro^ metastable states m1_c11 **(B)** and m2_c5 **(C)**. The structural analysis was performed using PyMOL (WL, 2002; Yuan et al., 2017).

The catalytic sites of these two states were then analyzed using PyMOL (WL, 2002; Yuan et al., 2017) to determine any differences in the position of the catalytic site residues compared to the M^pro^ reference protein structure (PDB: 6Y84; https://www.rcsb.org/structure/6Y84) (Figure 3A). The M^pro^ metastable state m1_c11 showed a reversed orientation of the side chains of residues C145 and N142. The residue C145 is a part of the catalytic dyad (residue C145 and residue H41) of M^pro19,45^ and is critical for the proteolytic activity of the protein (Figure 3B). Additionally, residue N142 is reported to form vdW interactions with the glutamine residue (Q) of the substrate peptide (AVLQSGFR) (Shaqra et al., 2022). The M^pro^ metastable state m2_c5 also showed a change in the side chain orientation of residue Q189 (Figure 3B). However, no discernable difference in the position of residues T25, G143, and E166 was observed. The M^pro^ metastable state m2_c5, on the other hand, showed a difference in the Cα atom positions and alterations in the side chain orientations of the catalytic site residues. Furthermore, the distances between residues T25, H41, C145, and E166 were observed to be decreased, while residues N142 and G143 appeared to be distant from these catalytic site residues. Importantly, residue Q189 showed a shift in its location and appeared to move away from the catalytic site, with its side chain pointing away from the bulk of the protein (Figure 3C). The side chain of residue Q189 is critical for forming a cavity that positions the P2 residue of substrate peptide, leucine (L), in case of N-terminal M^pro^ substrate cleavage site (NSP4-5 = AVLQSGFR) (Shaqra et al., 2022). The side chain and the backbone of residue Q189 have been reported to form van der Waals (vdW) and hydrogen bond (H-bond) interactions with all M^pro^ substrate cleavage site peptides except for NSP14-15 (not reported for NSP14-15 as of yet) (Chan et al., 2021; Shaqra et al., 2022).

**FIGURE 4:**
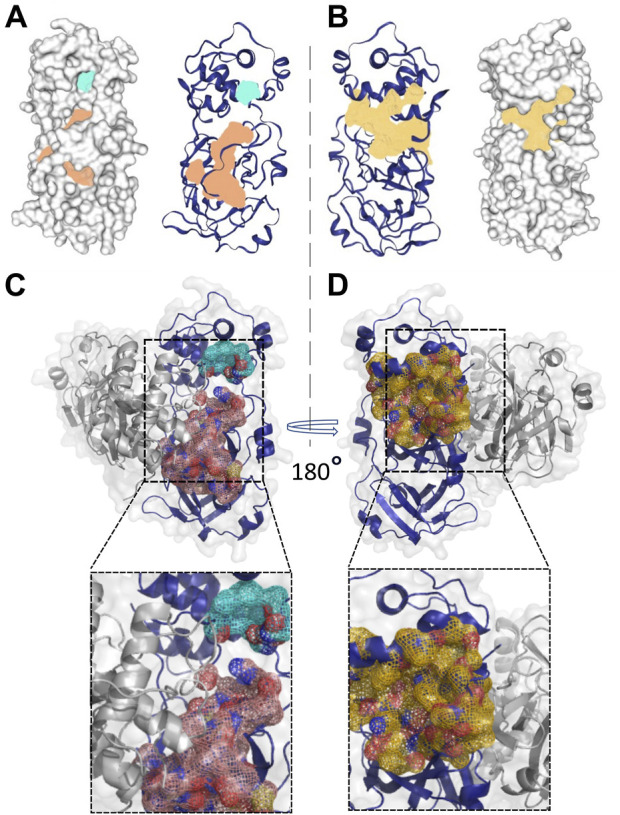
Location and shape of the two predicted potentially druggable allosteric sites in the M^pro^ metastable state m2_c5. **(A, B)** Surface (left panel) and cartoon (right panel) representation of two potential druggable allosteric sites in the M^pro^ metastable state m2_c5 with the shaded area representing allosteric site 1 (light orange, allosteric site 1 and light sea green, allosteric site1′) **(A)** and allosteric site 2 (pale yellow, allosteric site 2) **(B)** between domain II and domain III of m2_c5. **(C, D)** Cartoon and surface representations of the M^pro^ dimer structure showing the position of the potentially druggable allosteric sites 1 **(C)** and 2 **(D)**. Outsets in C and D show zoomed-in views of the potentially druggable allosteric sites 1 and 2 in the M^pro^ dimer structure. The allosteric site 1 is partly located at the dimer interface, while allosteric site 2 is located opposite to the dimer interface of M^pro^.

Based on the structural differences discussed above, we selected the M^pro^ metastable state m2_c5 as a metastable state having a deformed catalytic site for further studies. Next, we attempted to find a potential druggable allosteric site on this metastable state using the DoGSiteScorer tool (Volkamer et al., 2012; Volkamer et al., 2010), which is an automated pocket detection and analysis tool to predict the potential binding pockets and sub-pockets in protein structures (Volkamer et al., 2012). We selected potential binding sites based on the drug score (Volkamer et al., 2012), an output parameter with values ranging from 0 to 1, which provides an estimate of the druggability of the predicted sites (the higher the score, the more druggable the site is) (Volkamer et al., 2012). The druggability or drug score is calculated for each (sub)pocket using the linear combination of the three descriptors describing volume, hydrophobicity, and enclosure (Volkamer et al., 2012). Initially, the site with the highest drug score was selected as the putative allosteric site, which we named as allosteric site 1a. The allosteric site 1a contained 38 residues (list of residues is given in Table 1) with the drug score of 0.83, enclosure (ratio of site hull to surface grid points) of 0.09, and a depth of 26.21 Å (Figure 4A; highlighted in orange). In addition to this site, another site was detected by DoGSiteScorer (Volkamer et al., 2012), which we named allosteric site 1b. Located near the allosteric site 1a, this was of a relatively small size and had a low drug score (0.26) but had a high polar residue ratio of 0.56. In the subsequent *in silico* screening, we utilized a relatively large grid box that included allosteric sites 1a and 1b (together formed the allosteric site 1) to ensure a maximum number of hit identifications (Figure 4A; highlighted in turquoise). Importantly, we note that some of the residues in the predicted allosteric site 1 (residues G29, L115, R131, G149, D197, and E290) have been reported to be sensitive to mutation for the M^pro^ proteolytic activity (Flynn et al., 2022).

**TABLE 1:** Druggability analysis of the potential allosteric sites in M^pro^. Table showing the results of the druggability analysis and size and shape descriptors of the two potential allosteric sites predicted in the M^pro^ metastable state m2_c5 using DoGSiteScorer (Volkamer et al., 2012).

DoGSiteScorer analysis of the m2_c5 (M^pro^ State) for pockets								
Pocket		Residues	Drug score	Volume [Å³]	Surface [Å^2^]	Depth [Å]	Enclosure	Polar amino acid ratio
Allosteric site1	Allosteric site1a	M17, L27, N28, G29, L32, T45, F103, F112, V114-Y118, Y126 -R131, T135- G149, F159, H172, L177, D197, and E290.	0.83	910.98	1174.62	26.21	0.09	0.46
	Allosteric site1b	T199, T200, V204, Y237, N238, Y239, L268, L272, and L287.	0.26	115.84	226.81	9	0.17	0.56
Allosteric site 2		F3, R4, M6, V104, R105, I106, P108, G109, Q127, M130, R131, P132, F150, N151, I152, D 153, Y154, S158, C160, I200, E240, H246, G251, Q256, E290, S301, and V303-Q306.	0.8	1325.25	1713.21	19.15	0.16	0.34

In addition to the allosteric site 1, the DoGSiteScorer (Volkamer et al., 2012) analysis also predicted another potential allosteric site, which we named allosteric site 2, which showed the second-highest drug score of 0.8. This site comprises 30 residues (the list of residues is provided in Table 1) (Figure 4B; highlighted in pale yellow). Some of the key residues of the allosteric site 2, such as residues I200 and H246, were also reported by Günther et al. (2021) to play a role in the allosteric regulation of M^pro^ proteolytic activity. However, the M^pro^ metastable state m2_c5 appeared to be more accessible with much less enclosure (0.16) compared to the previously reported site (enclosure: 0.72) at the same location (Alzyoud et al., 2022).

Additionally, we determined the location of the predicted allosteric sites in the M^pro^ dimer to ensure that these sites are accessible for ligand binding in the dimeric structure of M^pro^, which is the catalytically competent form of the protein (Jin et al., 2020). This analysis revealed a relatively small portion of the allosteric site 1a to be located at the M^pro^ dimer interface.

Overall, the allosteric site 1 appeared to be accessible for ligand binding in the dimer form of M^pro^ (Figure 4C). On the other hand, the allosteric site 2 was located opposite to the dimer interface, making it completely accessible for ligand binding in the M^pro^ dimer (Figure 4D).

### Identification of high-affinity compounds targeting the potential allosteric site

Having determined a metastable state with a deformed catalytic site and a potential allosteric ligand binding site, we performed an *in silico* screening of a drug-like compound library (∼4.2 million) obtained from the ZINC15 database (Irwin and Shoichet, 2005). For this, we first performed site-specific docking using the idock algorithm (Li et al., 2012) with grid parameters specified for allosteric site 1 in M^pro^ to identify potential binders for this site. Following *in silico* screening, we selected 400 top-ranking compounds showing binding energies lower than −9 kcal/mol and proceeded with a step of blind docking using AutoDock Vina (Trott and Olson, 2010) to determine whether these compounds prefer binding to the predicted allosteric sites. Following the docking, we analyzed the binding poses of all the 400 compounds using PyMOL (WL, 2002;Yuan et al., 2017) and found them to bind to three distinct sites on M^pro^. These were the catalytic site and the potential allosteric sites 1 and 2. We then applied a selection criterion wherein we selected compounds that showed binding to the allosteric sites more than once in the nine binding poses generated in the blind, AutoDock Vina docking runs. Finally, we identified three compounds, namely, ZINC11696924, ZINC12383815, and ZINC4497834, having high affinity for the predicted allosteric sites and further analyzed them for their binding to the potential allosteric sites in M^pro^.

**FIGURE 5:**
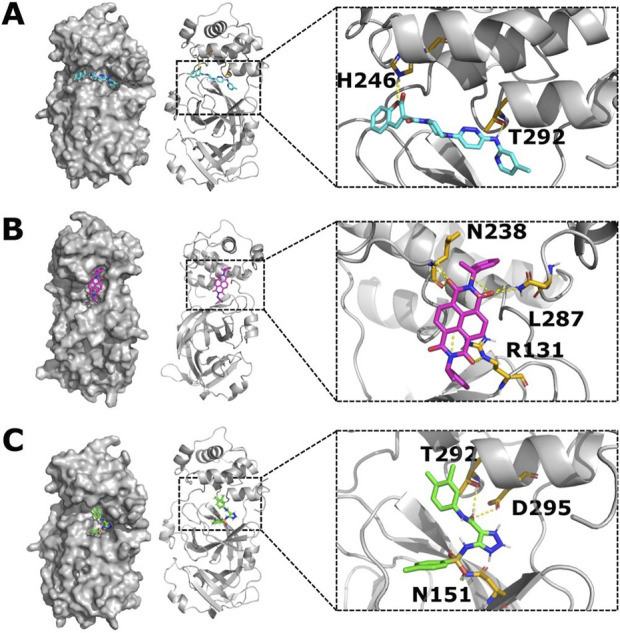
Top-ranking ligands (ZINC11696924, ZINC12383815, and ZINC4497834) are predicted to bind to the potential allosteric sites in the M^pro^ metastable state m2_c5. **(A–C)** Surface (left panel) and cartoon (right panel) representation of the M^pro^ metastable state m2_c5 showing docked ligands ZINC11696924 **(A)**, ZINC12383815 **(B)**, and ZINC4497834 **(C)**. Outsets show polar interactions between the ligands and M^pro^ residues identified using PyMOL (WL, 2002; Yuan et al., 2017) software (ZINC11696924 interacts with residues H246 and T292; ZINC12383815 interacts with residues N238, L287, and R131; and ZINC4497834 interacts with residues T292, D295, and N151) in the respective complexes.

First, the compound ZINC11696924 was found to bind to the allosteric site 2 in five docking poses and to the allosteric site 1 in only one docking pose out of nine docking poses. The top-ranking binding pose with a binding affinity of −9.9 kcal/mol showed polar interactions of ZINC11696924 with residues H246 and T292 in allosteric site 2 (Figure 5A). The residue H246 is one of the important histidine residues of M^pro^ and is crucial for the stability of the M^pro^ structure (Pavlova et al., 2021). On the other hand, the residue T292 has an allosteric effect on the catalytic activity of M^pro^ as mutation at this position with positively charged residues results in the loss of M^pro^ activity (Flynn et al., 2022).

The second compound ZINC12383815 showed binding to the allosteric site 1 in two out of the nine docking poses. It showed polar interactions with residues R131, N283, and L287 of M^pro^ with a top affinity of −10.0 kcal/mol (Figure 5B). Out of these three residues that showed an interaction with ZINC12383815, residue R131 plays a critical role in the structural plasticity and flexibility of the M^pro^ structure due to the formation of a salt bridge with residue D289 (Flynn et al., 2022; Bhat et al., 2022a).

The third compound ZINC4497834 showed binding to the allosteric site 2 in five out of the nine docking poses and revealed polar interactions with residues N151, T292, and D295 in M^pro^ with the top predicted affinity of −8.2 kcal/mol (Figure 5C). The residue D295 is one of the salt bridge-forming residues, and any substitution at this position leads to a complete loss of M^pro^ activity (Flynn et al., 2022; Kaptan et al., 2022). Additionally, it has been reported that the M^pro^ nanobody, N2B4, which acts as an allosteric inhibitor of the protease, also interacts with the residue D295 (Sun et al., 2022). Together, these suggest that the compound ZINC4497834 can allosterically impact the catalytic activity of M^pro^, given that it appears to bind to the potential allosteric site.

Overall, our *in silico* screening led to the identification of three potential high-affinity binders of the potential allosteric sites in M^pro^. ADME profiles of these three compounds, as predicted by SwissADME, were found to be suitable. In addition, the three compounds showed a good pharmacokinetic profile and druglikeness (Table 2). Although the compound ZINC4497834 did not appear to violate any druglikeness criteria with a good pharmacokinetic profile, the compounds ZINC11696924 and ZINC12383815 appeared to violate one of Lipinski’s rule and also appear to inhibit a few of the cytochrome-P450 enzymes (Table 2). The chemical structures of ZINC11696924, ZINC12383815, and ZINC4497834 are given in Supplementary Figure S1.

**TABLE 2:** Physiochemical properties, pharmacokinetics, and druglikeness of the top-ranking ligands. Table showing physiochemical properties, pharmacokinetics, and druglikeness of the top-ranking ligands predicted using the SwissADME web tool (Daina et al., 2017). TPSA, topological polar surface area; GI absorption, gastrointestinal absorption, BBB permeation, blood–brain barrier permeation; Pgp substrate, P-glycoprotein substrate.

Physiochemical properties			
Compound	ZINC11696924	ZINC12383815	ZINC4497834
Formula	C_24_H_22_N_6_O_3_	C_30_H_22_N_2_O_4_	C_18_H_19_N_5_O_3_S
Molecular weight	442.47	474.51	385.44
#Heavy atoms	33	36	27
#Aromatic heavy atoms	22	28	17
#Rotatable bonds	5	4	6
#H-bond acceptors	6	4	5
#H-bond donors	1	0	3
TPSA	104.46	78.14	125.22
Pharmacokinetics			
GI absorption	High	High	High
BBB permeant	No	No	No
CYP1A2 inhibitor	No	No	No
CYP2C19 inhibitor	Yes	Yes	No
CYP2C9 inhibitor	Yes	Yes	No
CYP2D6 inhibitor	No	No	No
CYP3A4 inhibitor	Yes	No	Yes
Pgp substrate	Yes	No	No
Druglikeness			
Lipinski #violations	0	1	0
Ghose #violations	1	1	0
Veber #violations	0	0	0
Egan #violations	0	0	0
Muegge #violations	0	0	0
Bioavailability score	0.55	0.55	0.55

### Stable binding of ZINC11696924, ZINC12383815, and ZINC4497834 to the potential allosteric sites in M^pro^

**FIGURE 6:**
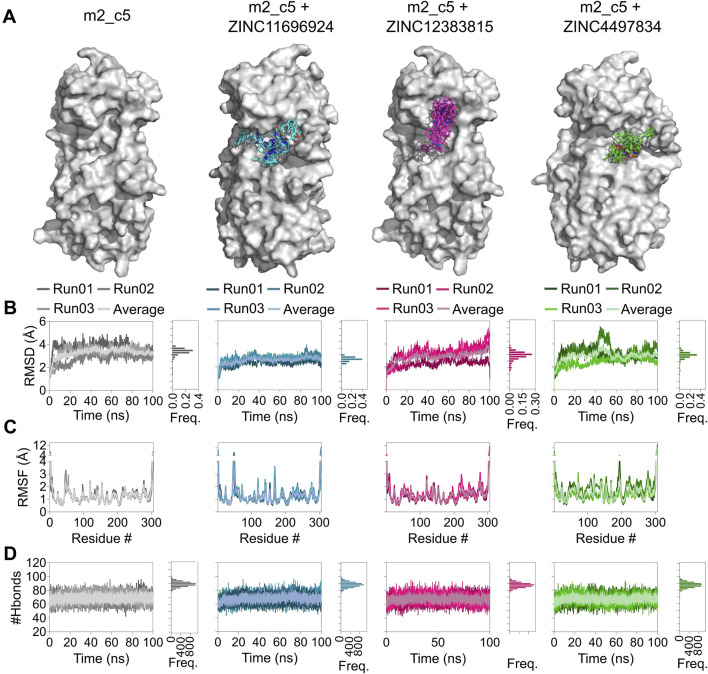
Altered structural dynamics of the M^pro^ metastable state m2_c5 in complex with the potential allosteric site binders ZINC11696924, ZINC12383815, and ZINC4497834. **(A)** Surface representation of the M^pro^ metastable state m2_c5 in the absence of ligand or in the presence of bound ligands ZINC11696924 (cyan), ZINC12383815 (magenta), and ZINC4497834 (green) showing orientations of the ligands obtained from 100-ns MD simulations, captured every 10 ns. **(B)** Graphs showing RMSD values (left panel) and frequency distribution of the RMSD values (right panel) of the M^pro^ metastable state m2_c5 in the absence of any ligand and in complex with ligands ZINC11696924 (cyan), ZINC12383815 (magenta), and ZINC4497834 (green) obtained from 100 ns MD simulations. **(C)** Graphs showing RMSF values of the M^pro^ metastable state m2_c5 in the absence of any ligand and in complex with ligands ZINC11696924 (cyan), ZINC12383815 (magenta), and ZINC4497834 (green) obtained from 100 ns MD simulations. **(D)** Graphs showing the number of H-bonds in the M^pro^ metastable state m2_c5 in the absence of any ligand (apo, gray) and in complex with ligands ZINC11696924 (cyan), ZINC12383815 (magenta), and ZINC4497834 (green). Data shown are obtained from three independent, 100 ns long, all-atom, explicit solvent MD simulations.

To investigate the binding stability of the three identified compounds to their respective allosteric sites, we performed three independent 100 ns, all-atom, explicit solvent, MD simulations of the apo M^pro^ metastable state m2_c5 and M^pro^ metastable state m2_c5 in complex with high-affinity allosteric binders, using NAMD2 (Phillips et al., 2005). Analysis of the MD trajectories showed that the ligands were retained in the respective allosteric sites across the 100 ns simulation time (Figure 6A). However, the change in orientation of the compounds was observed in the respective allosteric sites (Figure 6A). Additionally, RMSD analysis showed a general decrease in the structural dynamics of the protein as a result of ligand binding to the allosteric site (Figure 6B). Specifically, RMSF analysis of the M^pro^ metastable state m2_c5 bound with the compound ZINC11696924 showed an increase in the flexibility of a region in the middle of domain I (residues ranging from 46 to 51) and at the end of domain II (residues ranging from 178 to 199), respectively. The residues ranging from 46 to 51 are a part of the S2 subsite of the M^pro^ catalytic site (Parmar et al., 2022). Moreover, the end of the domain II (residues ranging from 178 to 199) region contains an alpha helix and a loop that connects domain II to domain III (Parmar et al., 2022) of the M^pro^ metastable state m2_c5, and the observed increase in the flexibility of this region may affect the catalytic activity of M^pro^. On the other hand, the M^pro^ metastable state m2_c5 in complex with ZINC12383815 showed a decrease in RMSF values in the region containing residues ranging from C44 to D56. We note that the residue S46 has been shown to interact with the substrate peptide, while the residue M49 has been reported to form the ‘lid’ of the S2 hydrophobic subsite in the M^pro^ catalytic site (Parmar et al., 2022). Finally, the M^pro^ metastable state m2_c5 bound with the compound ZINC4497834 showed an overall increase in the RMSF values compared to that of the apo M^pro^ metastable state m2_c5 (Figure 6C), except for a decrease in the RMSF values of residues ranging from T45 to L50. Importantly, there was an increase in the flexibility of a region encompassing residues ranging from T135 to C145, which contains two catalytic site residues N142 and G143 and one M^pro^ catalytic dyad (Puhl et al., 2019) residue C145 (Figure 6C). The observed RMSF differences in the M^pro^ metastable state in apo and in complex with the compounds ZINC11696924, ZINC12383815, and ZINC4497834 suggest an altered structural dynamics of the M^pro^ metastable state m2_c5 upon binding to potential allosteric binders. Contrary to the differences observed in the RMSF analysis, intra-protein hydrogen bond (H-bond) analysis did not reveal any large differences with the average number of H-bonds of 69 ± 4, 69 ± 4, 67 ± 4, and 67 ± 4 for the apo M^pro^ metastable state m2_c5 and those that are bound to ZINC11696924, ZINC12383815, and ZINC4497834, respectively (Figure 6D).

**FIGURE 7:**
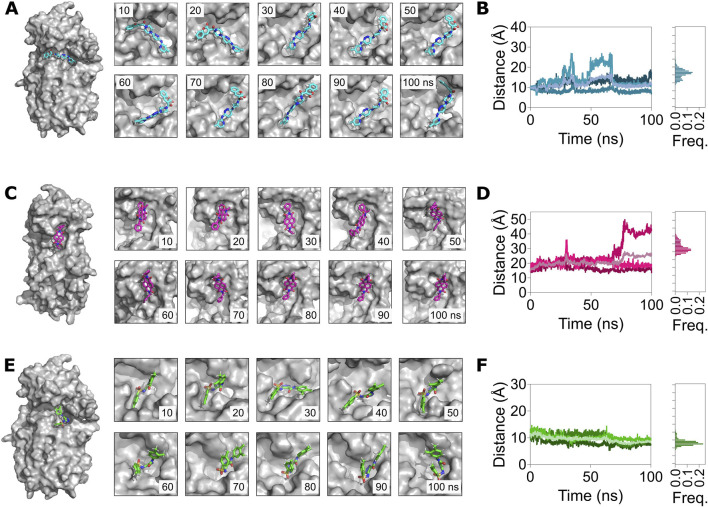
MD simulations reveal a stable binding of the allosteric site binders ZINC11696924, ZINC12383815, and ZINC4497834 with the predicted allosteric sites in the Mpro metastable state m2_c5. **(A,C,E)** Left panels: surface representation of the Mpro metastable state m2_c5 bound to ZINC11696924 **(A)**, ZINC12383815 **(C)**, and ZINC4497834 **(E)**. Right panels: snapshots of the allosteric site complex with ligands ZINC11696924 **(A)**, ZINC12383815 **(C)**, and ZINC4497834 **(E)** captured every 10 ns of a 100 ns MD simulation trajectory. **(B,D,F)** Left panels: graphs showing the center-of-mass distance between ligand interacting residues in the pocket (H246 and T292) and ZINC11696924 **(A)**, residues (N238, L287, and R131) and ZINC12383815 **(C)**, and residues (N151, T292, and D295) and ZINC4497834 **(E)**. Right panels: frequency distribution of the measured distance of the respective Mpro metastable state m2_c5 with allosteric binders. Data shown are obtained from three independent 100 ns long, all-atom, explicit solvent MD simulations.

We then assessed the distance of the compounds from their interacting residues to determine if they remained bound to the potential allosteric sites over the 100 ns trajectories (Figure 7A). Overall, this analysis revealed that all three compounds remained bound to their allosteric sites in M^pro^. A closure inspection revealed that the distance of the compound ZINC11696924 with its interacting residues H246 and T292 increased from 10 Å, measured at the beginning of the simulation, to approximately 13 Å over time (mean ± SD, 13 ± 2 Å) (Figure 7B). However, the distance of the compound ZINC12383815 with its interacting residues N238, L287, and R131 largely remained the same, with some increases observed during the initial stages of the simulations (27 ± 3 Å) (Figures 7C, D). On the other hand, the distance of the compound ZINC4497834 with its interacting residues N151, T292, and D295 decreased during the course of 100 ns MD simulations (8 ± 1 Å) (Figures 7E, F).

**FIGURE 8:**
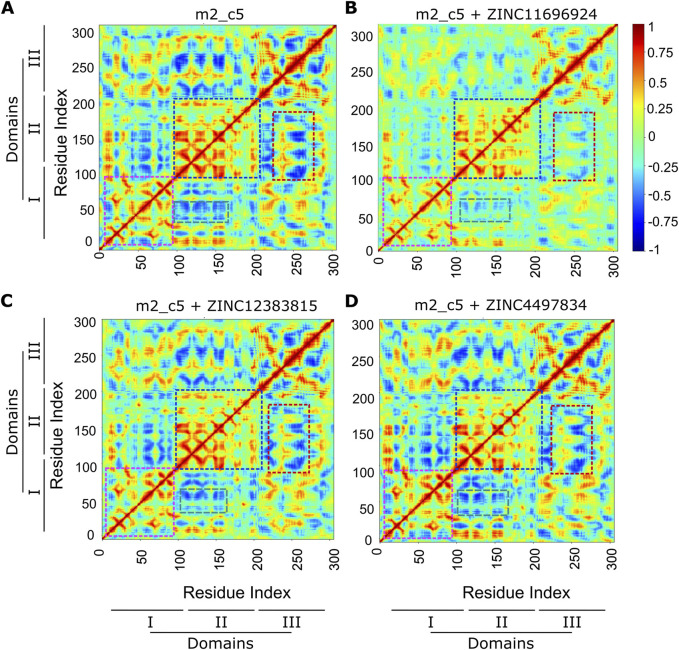
Altered cross-correlated motions of the M^pro^ metastable state m2_c5 in complex with ligands ZINC4497834, ZINC11696924, and ZINC12383815. **(A–D)** Heat maps showing average inter-residue dynamic cross-correlation (DCC) values of the M^pro^ metastable state m2_c5 in the apo **(A)** and in complex with ZINC11696924 **(B)**, ZINC12383815 **(C)**, and ZINC4497834 **(D)** obtained from three independent, 100 ns long MD simulations. Regions showing differences in DCC between the M^pro^ metastable state m2_c5 in the apo **(A)** and in complex with ZINC11696924 **(B)**, ZINC12383815 **(C)**, and ZINC4497834 **(D)** are highlighted with color boxes. Pink boxes, DCC of M^pro^ domain I residues; blue boxes, DCC of M^pro^ domain II residues; red boxes, DCC between M^pro^ domain III and II residues; green boxes, DCC between M^pro^ domain II and I residues.

Having found a generally stable binding of the compounds to the M^pro^ metastable state m2_c5, we then analyzed the MD simulation trajectories for any changes in correlated motions of the residue in the protein upon binding the potential allosteric binders using the dynamic cross-correlation (DCC) algorithm available as a part of the MD-TASK suite of Python scripts (Brown et al., 2017). This analysis revealed a general reduction in cross-correlated motions between residues in the intra-domain regions of the M^pro^ metastable state m2_c5 in the presence of all three potential allosteric binders (Figure 8). Specifically, the DCC values of residues in domain I (highlighted using pink boxes) and domain II (highlighted using blue boxes) were high in the apo M^pro^ metastable state m2_c5 but showed a reduction when the protein was bound to either of the three compounds ZINC11696924, ZINC12383815, and ZINC4497834 (Figures 8A–D), suggesting that the binding of these ligands results in a decrease in correlated motions in the individual domains of the protein. Aside from the intra-domain cross-correlation, residues in domain III showed a negative cross-correlation with residues in domains I and II. Interestingly, these negatively correlated motions appeared to be decreased in the presence of the compound ZINC11696924 (Figures 8A, B). Similarly, a reduction in the negatively correlated motions of residues in domain III with those in domain II was observed in the presence of the compounds ZINC12383815 and ZINC4497834 (Figures 8A, C; highlighted using red boxes). Additionally, an increase in the negatively correlated motions was observed between residues in domain III with those in domain II in the presence of compound ZINC4497834 compared to the apo M^pro^ metastable state m2_c5 (Figures 8A, D; highlighted using green boxes). Overall, these results are suggestive of an effect of these potential allosteric binders on the structural dynamics of the M^pro^ metastable state m2_c5 that might lead to an alteration in the M^pro^ proteolytic activity.

**FIGURE 9:**
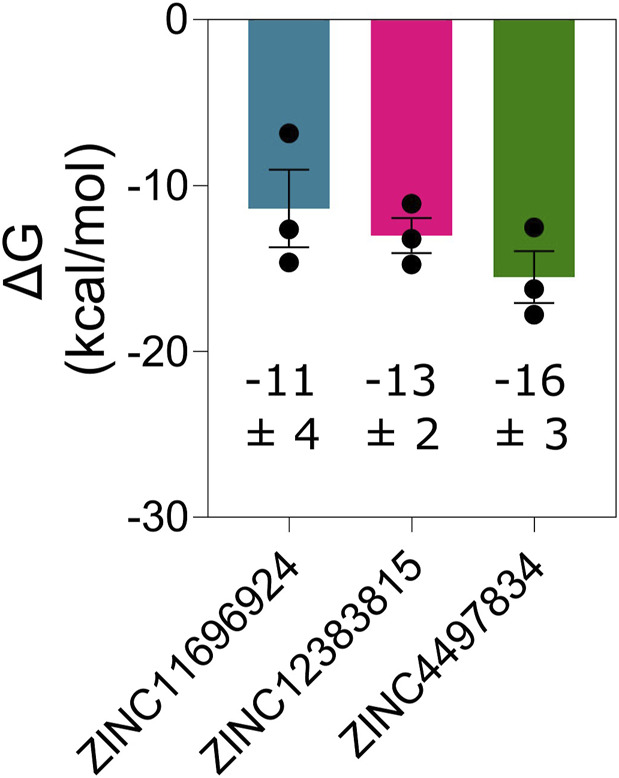
M^pro^ metastable state m2_c5 binds ligands ZINC11696924, ZINC12383815, and ZINC4497834 at the allosteric site with high affinity. Graph showing free energy change of binding (ΔG) of the M^pro^ metastable state m2_c5 with the indicated predicted allosteric site binders. Data shown are mean ± SD obtained from three independent, 100 ns long MD simulations determined at an interval of 20 ns.

Next, we determined the binding affinity of the potential allosteric binders through the calculation of free energy change of binding of the compounds ZINC11696924, ZINC12383815, and ZINC4497834 with the M^pro^ metastable state m2_c5 using MD simulation trajectories and the CaFE 1.0 tool (Liu and Hou, 2016) in conjunction with VMD (Humphrey et al., 1996). This analysis revealed generally high-affinity binding of the potential allosteric compounds with free energy change of binding (∆G) values of −11 ± 4, −13 ± 2, and −16 ± 3 kcal/mol for ZINC11696924, ZINC12383815, and ZINC4497834, respectively (Figure 9).

### ZINC4497834 inhibits the M^pro^ proteolytic activity *in vitro*

**FIGURE 10:**
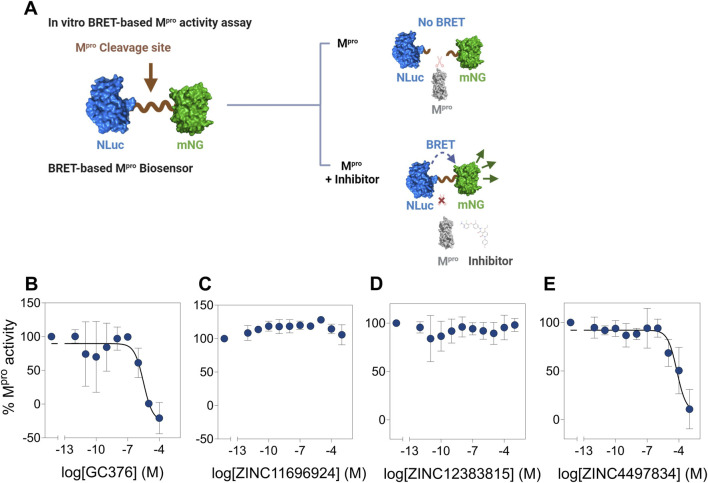
Potential allosteric site binding ligand ZINC4497834 inhibits M^pro^ activity *in vitro*. **(A)** Schematic representation showing the working of a BRET-based M^pro^ biosensor containing the M^pro^ N-terminal autocleavage sequence between mNG (BRET acceptor) and NLuc (BRET donor). The biosensor shows high BRET (ratio of mNG fluorescence and NLuc bioluminescence) in the absence of M^pro^, while M^pro^-mediated cleavage results in the decrease in the BRET due to physical separation of mNG and NLuc. However, in the presence of an inhibitor, cleavage of the biosensor will be abrogated and result in the maintenance of high BRET. **(B–E)** Graphs showing the percentage M^pro^ proteolytic cleavage activity at various concentrations of the covalent inhibitor GC376 **(B)** and potential allosteric ligands ZINC11696924 **(C)**, ZINC12383815 **(D)**, and ZINC4497834 **(E)**. All BRET ratios in a given experiment were first normalized with those obtained in the absence of M^pro^, and the normalized BRET ratios were used for determining % M^pro^ activities. Data shown are mean ± SD obtained from two **(B)** or three **(C–E)** independent experiments.

Having established stable and high-affinity binding of the three potential allosteric binders using computational methods, we aimed to determine the impact of these compounds (ZINC11696924, ZINC12383815, and ZINC4497834) on the proteolytic activity of M^pro^ in an *in vitro* assay. For this, we utilized our recently reported BRET-based M^pro^ biosensor (Geethakumari et al., 2022a). The biosensor consists of mNG (BRET acceptor) on the N-terminal side, M^pro^ N-terminal auto-cleavage sequence (AVLQSGFR) in the middle, and NLuc luciferase (BRET donor) on the C-terminal side. It shows high BRET in the absence of M^pro^ activity due to the proximity of NLuc and mNG, while it shows a reduction in BRET in the presence of M^pro^ activity due to the physical separation of NLuc and mNG (Figure 10A). We incubated lysates prepared from HEK 293T cells expressing the biosensor with a recombinantly purified M^pro^ with increasing concentrations of the potential allosteric compounds and monitored BRET after addition of the NLuc luciferase substrate (Figure 10). Incubation of M^pro^ with the known M^pro^ covalent inhibitor, GC376 (Geethakumari et al., 2022a; Sharun et al., 2021; Lu et al., 2022; Luan et al., 2023), resulted in a concentration-dependent decrease in M^pro^ activity with an IC_50_ value of 4.3 ± 5.5 µM (Figure 10B). Importantly, incubation of M^pro^ with the compounds ZINC11696924 and ZINC12383815 did not show any notable decrease in its activity (albeit the compound ZINC11696924 appeared to show some increase in M^pro^ activity at lower concentrations) (Figures 10C, D). On the other hand, incubation of M^pro^ with the compound ZINC4497834 resulted in a concentration-dependent decrease in its activity with an *IC*_50_ value of 43 ± 39 µM (Figure 10E), which is similar to the *IC*_50_ value (25.16 µM) of the recently reported allosteric inhibitor AT7519 (Günther et al., 2021). Importantly, AT7519 (Günther et al., 2021) was shown to bind to allosteric site 2, which is also the site where the compound ZINC4497834 was found to be stably bound in our computational studies (Figure 10E) (Günther et al., 2021). Together, these results suggest that the compound ZINC4497834 inhibits SARS-CoV-2 M^pro^, likely through an allosteric mechanism.

**FIGURE 11:**
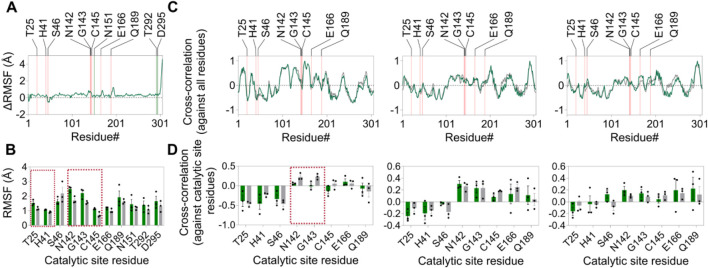
Altered structural dynamics of the M^pro^ metastable state m2_c5 induced by ZINC4497834. **(A)** Graphs showing the change in average RMSF (ΔRMSF) of residues in the M^pro^ metastable state m2_c5 in the presence of bound ZINC4497834 (orange and green lines indicate the location of catalytic site residues and ligand interacting residues on the *x*-axis, respectively). ΔRMSF values were determined by subtracting the average RMSF values of residues in the apo M^pro^ metastable state m2_c5 from the average RMSF values of residues in the M^pro^ metastable state m2_c5 bound to ZINC4497834. **(B)** Graph showing the RMSF(Å) of catalytic site residues and ZINC4497834-interacting residues in the apo (gray bars) and ZINC4497834-bound (green bars) M^pro^ metastable state m2_c5. **(C)** Graphs showing the average DCC values of residues N151 (right panel), T292 (middle panel), and D295 (left panel) against all residues in the protein (orange lines indicate the location of catalytic site residues on the *x*-axis). **(D)** Graphs showing the DCC values of residue N151 (left panel), T292 (middle panel), and D295 (right panel) with catalytic site residues. The dashed red boxes in B and D highlight the residues with significant difference in RMSF **(B)** and DCC values **(D)** between apo and the ZINC4497834-bound M^pro^ metastable state m2_c5, respectively. Data shown are obtained from three independent, 100 ns-long MD simulations.

After establishing high-affinity binding and subsequent inhibition of M^pro^ activity by the compound ZINC4497834 in the BRET-based *in vitro* assay (Figures 5C, 6, 7E), we aimed to explore the probable mechanism underlying this inhibition. For this, we first revisited the MD simulation trajectories and analyzed the difference in the average RMSF values of each residue in the apo- and compound ZINC4497834-bound form of the M^pro^ metastable state m2_c5. This analysis revealed an overall increase in the fluctuations of all the residues of the M^pro^ metastable state m2_c5 bound with ZINC4497834 (Figures 11A). Importantly, five catalytic site residues T25 (*p* = 0.043), H41 (*p* = 0.013), N142 (*p* = 0.012), G143 (*p* = 0.039), and C145 (*p* = 0.020) showed a significant increase in the RMSF in the M^pro^ metastable state m2_c5 bound with ZINC4497834 (Figure 11B). Interestingly, however, there was no significant difference in the fluctuations of the ZINC4497834-interacting allosteric site 2 residues N151, T292, and D295 in the apo and ZINC4497834-bound M^pro^ metastable state m2_c5 (Figure 11B). Second, we performed a closure inspection of the dynamic cross-correlation values of the ZINC4497834-interacting allosteric site 2 residues N151, T292, and D295 with all other residues in the apo and ZINC4497834-bound M^pro^ metastable state m2_c5 (Figure 11C). This analysis revealed a significant decrease in the positive cross-correlated motions of N151 with catalytic site residues N142 (*p* = 0.034) and G143 (*p* = 0.04) (Figure 11D, left panel, highlighted with red box). Third, we would like to note that the allosteric inhibitor AT7519, identified through X-ray screening by Günther et al. (2021), also binds to the allosteric site 2 that we have identified here and interacts with the residue D153, which is close to residues N151 and D295 (Günther et al., 2021). Finally, a comprehensive deep mutational scanning of M^pro^ by Flynn et al. (2022) revealed that residue D295 has a low mutation tolerance (Flynn et al., 2022). Together, these provide a probable mechanism underlying the inhibition of M^pro^ due to the binding of ZINC4497834 to the predicted allosteric site in the protein.

## Conclusion

To conclude, we performed a structural analysis of metastable states of M^pro^ identified from MD simulation and identified an M^pro^ metastable state (m2_c5) having a deformed catalytic site based on its affinity for the M^pro^ N-terminal auto-cleavage sequence. Furthermore, we identified two potential druggable allosteric sites on the M^pro^ metastable state m2_c5 and performed *in silico* screening (molecular docking) of a library of drug-like compounds obtained from the ZINC15 database, which resulted in the identification of three potential high-affinity allosteric site-binding compounds. Additional computational analysis, MD simulations, revealed a stable binding of selected compounds to their respective allosteric sites and a change in the M^pro^ metastable state m2_c5 structural dynamics upon binding to high-affinity allosteric site compounds. Importantly, one of the three selected compounds, ZINC4497834, inhibited SARS-CoV-2 M^pro^ activity in BRET-based *in vitro* assay, thus suggesting that this compound could act as an allosteric inhibitor of SARS-CoV-2 M^pro^. We envisage that this compound can be taken further down the drug discovery pipeline as an allosteric inhibitor of SARS-CoV-2 M^pro^ after further research, including mutational analysis, live cell, and *in vivo* experiments. Moreover, the allosteric sites identified in this study can be targeted for the discovery of highly potent M^pro^ allosteric inhibitors.

## Data Availability

The original contributions presented in the study are included in the article/Supplementary Material; further inquiries can be directed to the corresponding author.
