# Supplementary material for: A potential allosteric inhibitor of SARS-CoV-2 main protease (Mpro) identified through metastable state analysis
Source: Front Mol Biosci. 2024 Sep 6;11:1451280. doi: 10.3389/fmolb.2024.1451280 (PMC11413593; doi:10.3389/fmolb.2024.1451280)
Supplement: Supplementary file 1 [file Image1.pdf]

Supplementary Figures:

ZINC11696924

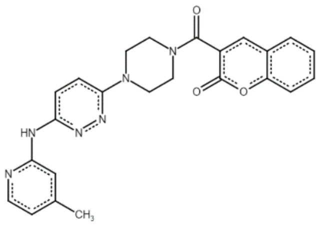

ZINC12383815

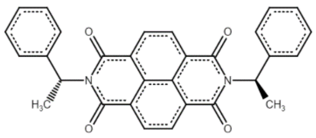

ZINC4497834

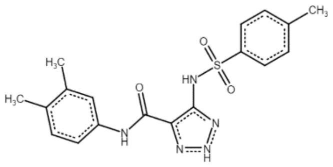

Supplementary Figure 1. Chemical structures of ZINC4497834, ZINC11696924 and ZINC12383815. *The structures were drawn using chemical sketch tool (*https://www.rcsb.org/chemical-sketch*) using SMILES**.***
